# British Romantic Generalism in the Age of Specialism, 1870–1990

**DOI:** 10.1093/shm/hkv103

**Published:** 2015-10-14

**Authors:** Stephen T. Casper, Rick Welsh

**Keywords:** generalism, general practice, specialisation, history of neurology, neurologists, history of medicine, nineteenth-century medicine, twentieth-century medicine, British medicine

## Abstract

This essay explores the impact of ‘generalism’ and ‘general practice’ on the specialisation of British medicine using the case of neurology in Britain to reveal characteristics of British ‘generalist medical culture’ from 1870 to 1990. It argues that ‘generalism’ represented a particular epistemological position in Victorian medicine, one that then created a natural bridge between science and medicine over which almost all physicians and scientists were comfortable walking. The legacies of that Victorian ‘generalist preference’ exerted an enduring impact on the specialisation process as physicians experienced it in the twentieth century and as this case of neurology reveals so clearly. Neurologists and general physicians would still be arguing about the relative merits of a general medical education into the 1980s. By then, however, the emergence of government bodies promoting specialist labour conditions would have rendered the process seemingly inexorable.

## Introduction

Ever since Christopher Lawrence's pioneering identification of a patrician sensibility among medical practitioners in Victorian and Edwardian Britain, historians and sociologists of medicine have routinely placed what we shall call for brevity the modernisation of British medicine into the context of the culture it ostensibly replaced.^1^ The emergence of modernisation, including for example the role of centralisation, regulation, specialisation and standardisation, have provided many historical studies of medicine from late-Victorian Britain to the contemporary period with their underlying teleology. Yet, just as the ‘patrician sensibility’ did not disappear with the advent of medical modernity, the structures and processes of nineteenth-century medicine did not cease exerting palpable influence under the pressures of medical modernisation.^2^ As modernisation occurred, other patterns of medical culture persisted in appreciable ways. In part, this persistence explains differential experiences of patient care and medical labour in rural and urban general practice, the equitable distribution of health resources across the country, and the slow transfer of scientific, medical, and technical knowledge and skill from the confines of the medical teaching hospital to less august sites of medical work.

In nineteenth-century British medical culture a peculiar relationship had formed between the life sciences and medicine, a relationship filled with Romantic-era resonances. Moreover, the relationship lasted well-beyond the interwar period of the twentieth century.^3^ This relationship conjured at once the Romantic ideal of the unity of knowledge and faith in the progress of medicine and science. It recalled heroic gentleman of science as well. Illustrious figures populated this imagined world. There Charles Darwin and Michael Foster found ready company with the likes of John Hunter, Edward Jenner, Astley Cooper, Joseph Lister, John Hughes Bennet or Thomas Clifford Allbutt. There was also material evidence for this imagined world in the real one; ‘Fellowship’ in the Royal Society connected in a formidably Carlylian way individual discovery to unified, progressing science; ‘Fellowship’ in one of the Royal Colleges implied acumen in a range medical subjects.

British physicians and scientists therefore aspired to ‘generalism’, an ethic that held high competence and excellence in many medical and scientific subjects to be the calling of medicine—a definition that shall be used throughout this essay. ‘Generalists’ viewed discoveries in the natural and human sciences as demonstrating truths of transcendental significance; in medical practice they emphasised ‘general clinical observation’ even as they eschewed the laboratory, and in the British hospital they ‘reigned supreme’ until the second decade of the twentieth-century.^4^ For them, achievements of universal value in science and medicine defined excellence in medical practice and research, and thus much that was characteristic of British medico-scientific culture ranging from the Cambridge Natural Sciences Tripos to the creation of the National Institute for Medical Research captures these aspirations.^5^

At the same time a counter-trend developed. Specialisation, discipline formation and the steady appearance of the division of labour characteristically defined the modern appetite to ‘divide and conquer’.^6^ As social processes these developments were quintessential to the cultural experience of late industrial society's upheavals, instabilities, and experience of steady transformation (which many diagnosed as evidence of social disintegration).^7^ Specialisation foreshadowed advancing bureaucratisation as well, and it thus sat comfortably beside such other innovations as double-entry bookkeeping in the hospital and American-styled Taylorist efficiency in the factory.^8^ Thus, though the Romantic impulse in Britain inclined towards unitary visions of the progress of science and medical practice, there was a simultaneous, inexorable process of social reification that brought new categories of labour (specialties) into existence that possessed no meaningful historical counterpart.^9^ To be modern, it seems, was to be divided.

As is well known, many British medical practitioners' responses to these trends took the form of vehemence towards specialisation and its implications.^10^ But for all of that, their resistance to specialisation in Britain (and elsewhere) remains a past curiosity, a seemingly reactionary response to modernity analogous perhaps to William Morris' hostile if famous reply with *News from Nowhere* (1890) to Edward Bellamy's *Looking Backwards* (1887).^11^ There is, accordingly, ample opportunity to consider the multi-faceted dimensions of British resistance to specialisation and to do so in ways that delve somewhat deeper than exploration of the rhetoric against it.^12^

There are many reasons for believing that even as specialisation became a reality of British medical culture, a culture of medical generalism left a concomitant impression. William Osler's appointment as Regius Professor of Medicine at Oxford, the formation of such organisations as the Royal Society of Medicine and the Association of Physicians of Great Britain and Ireland, the persistent popularity of the medical magazine *The Practitioner*, the continual demand placed on medical schools to train students to be competent general practitioners, and even the notion that there should be such a thing as ‘general medicine’ can all be construed as signs that there was much underpinning generalist attitudes. Being a good general physician did not denote superficiality; neither for that matter did it imply knee-jerk dismissal of specialist knowledge. Rather, to competent general physicians, specialist knowledge meant transferable, usable knowledge—for students, general practitioners, physicians and surgeons, for medical lore *writ large*. Such knowledge could sell medical monographs and journal subscriptions, and for that matter could justify membership in some societies with special subjects, as, for instance, obstetrics and gynaecology.

The formation of the specialty of neurology in Britain provides a particularly clear means of examining the persistence of generalism even as specialisation became a reality.^13^ The study of the diseases of the nervous system and their treatment in the nineteenth century was a pursuit carried on by eminent physicians and scientists (often the same person), many of whom fashioned neurological knowledge in universalistic terms and regarded their subject as the logical development of their general clinical acumen and *vice versa*. For such physicians and scientists with interests in nervous and mental diseases and in the function of the nervous system, it became a practical commonplace for them to hold forth in published writings and lectures, as well as private conversations, on a range of sociological, anthropological, psychological and philosophical matters, not least of which was the philosophy of mind.^14^ Often at best agnostic about the process of specialisation, they regarded their studies and practice as specialised only insofar as the nervous system could be construed as integrating the whole of the body and mind together.^15^ Their clinical method, precise and exacting, was deemed not only difficult, but also of general clinical worth and productive of scientific progress.^16^ In a lecture in 1900 before the Nottingham Medical Society, Frederick W. Mott offered what was for then a typical view of the role played by the nervous system that captures all such themes:

The nervous system not only serves in maintaining the relation of the individual to his external environment in response to present or past stimuli from without, but also serves to correlate all the internal activities of the tissues and organs of the body by stimulating here, inhibiting there, and distributing the blood to the tissues in proportion to their activity. The nervous system is enabled to control the activities of all the tissues of the body by impressions conveyed to and from them by nerve fibers to and from various centres; but we also know that the nerve centres themselves are bio-chemically sensitive to the state of the blood occasioned by an organ which is diseased, when all the nervous paths from the organ have been interrupted.^17^

In one sense, of course, all-encompassing-views like Mott's existed against a background of competition among specialists.^18^ Yet physicians with interests in the nervous system could have easily expressed similar conflicts with general medicine and general practice.^19^ That they did not until the interwar period, and that they thereafter did so half-heartedly, is a significant indicator that for them specialisation entailed real trade-offs. In what follows, we shall draw upon manuscripts and published sources salient to the development of neurology to describe the culture of generalism that permeated the British medical landscape before the introduction of the National Health Service. We shall then consider the ways in which that culture left a significant imprint on the development of specialisation as it was practised within the health service after 1948.

## The Generalist Reaction, 1858–1914

Rosemary Stevens has observed that the period following the Medical Act of 1858 brought increased specialisation in the hospitals and private practice in Britain.^20^ The Act, which created the Medical Directory and the General Medical Council, however, followed the pattern of resistance to specialisation that so marked Britain's medical scene throughout the nineteenth century. The registry of practitioners, for example, did not acknowledge specific specialties, and physicians and general practitioners vigorously resisted medical specialisation throughout the period.^21^ Medical Consultants greeted specialisation with similar scepticism.^22^ Resistance to specialisation, however, did not prevent them from actively working in specialist hospital environments.^23^ In 1899, 195 physicians worked in London general hospitals, and only 31 of them did not also hold a position in a specialist hospital.^24^

Resistance to thoroughgoing specialisation developed for several reasons. Nineteenth-century hospitals—poor-law, county, general, voluntary and even specialist ones—throughout Britain were often stretched thin in terms of resources.^25^ Their chief sources of income were benefactions and subscriptions, although increasingly by the early twentieth century grants, State funds, small payments and contributory schemes were also present.^26^ Altering the arrangements of hospital wards by creating specialist departments ensured increasing expense. Increases in staff, and therefore costs, were a predictable consequence of medical specialisation. There were other reasons too. Hospital administrators and physicians alike worried that increasing the specialised training for medical students would complicate teaching and further overload an already unwieldy medical curriculum. Experience of the general wards, many felt, should mirror the chaos medical students would encounter in general practice.^27^

There was, too, a further motive. As Abel-Smith described, some felt that general practitioners sent patients to consultant's private practices when they could afford the consultant's fee but not necessarily the costs of hospitalization, in this way ensuring their patients preferential treatment through ‘queue-barging’ ahead of patients in out-patient hospital clinics. Such practices advantaged general practitioners and also the ‘monopoly value’ of Consultants' honorary appointments, and implied, or so we infer, that the advent of hospital beds delimited to specialist cases in special departments implied a corollary decrease (albeit not necessarily a one to one decrease) in the amount of beds available for general cases.^28^

Of course, as Stevens and Weisz have made so clear, resistance to specialisation in Britain did not prevent specialisation. Specialists could be prominent figures. Specialist hospitals and specialist private practices had been established even early in the nineteenth century. Stevens describes 128 specialist hospitals scattered across England and Wales by 1900, with those in London designated ‘centres of specialist teaching and research’.^29^ At the same time, there was high turnover in medical labour in special and general hospitals.^30^ Junior appointments in all hospitals were a means of remaining employed within the medical hierarchy until elected to the status of ‘Assistant’ or ‘Full Physician’ within a larger hospital—an appointment that was rarely a specialist one until the interwar period.^31^ Terms of junior appointments were brief, and doctors and surgeons in their early careers often held many different appointments simultaneously. Rising to the honorary status of ‘Full Physician’ in a voluntary hospital was viewed as the best path towards growing a large private practice, and individuals' circumstances along that career path, and their appointments in specialist hospitals as part of that journey, was often more happenchance than purposefully sought.

Medical societies followed these trends, too.^32^ Early nineteenth-century charitable voluntary associations had established medical care centres for the poor and elderly alike, and one effect of such voluntary associations was that growing numbers of patients began entering hospitals, prompting the formation of new ones.^33^ Such organisations sought to employ respectable practitioners, and membership in medical societies, especially those founded in provincial centres, provided some practitioners with much-sought legitimacy.^34^ By the time the Nottingham Medico-Chirurgical Society celebrated its centenary (1928), Humphrey Rolleston (1862–1944) could consequently observe that ‘the uses of a medical society were educational, for unity, peace, and friendship, and in certain circumstances for combined action in medico-political crises’.^35^

If unity and collegiality were mantras, the division that medical specialists represented in the profession became ever-more commonplace, especially in the rural Victorian world.^36^ But for would-be specialists, research and communication before their peers in general medical societies, as well as the ability to discourse on all medical topics with them, were essential means of demonstrating legitimate membership in a medical community. In fact, adopting a specialty often meant primarily further enhancing an income already generated by general practice. For these reasons medical practitioners brought their own research into public discourse, and thus did such societies become places of ‘friendly discourse’ and ‘agreeable refuge from the daily anxieties of medical practice’.^37^ For these reasons, too, specialist medical societies were not always aimed specifically towards communities of self-identified specialists. The Edinburgh Obstetrical Society, established in 1840 for example, ‘effectively remained for many years a society open to general practitioners’.^38^

These trends reached their zenith with the formation in 1907 of the Royal Society of Medicine and also the Association of Physicians of Great Britain and Ireland. While the Royal Society of Medicine had individual specialist sections ranging from neurology to medicine, its founders intended it to function as an umbrella organisation for former specialist societies. Many of London's hitherto autonomous specialist societies thereby gave up some autonomy in order to gain other measurable rewards through consolidation (a wider financial basis, central headquarters for operations and meetings, and a journal constituted powerful motives). Similarly, the Association of Physicians, which was founded in the same year and promoted itself as an organisation focused on ‘internal medicine’, cultivated members who dedicated themselves to medical research and teaching. In other words, although both organisations seemingly moved in ways embracing more specialisation and not less, both organisations ultimately sought to curtail the fragmentation caused by specialisation.

Members of the Association of Physicians were teachers of medicine and medical researchers. It is therefore noteworthy that their members were those most involved in the creation of both the Royal Society of Medicine and the Association of Physicians, and that they did much to bolster those societies' subsequent scientific proceedings. Richard Douglas Powell, as a leading figure in the creation of both societies, was perhaps exceptional. But like him the founders of the Association of Physicians, which included such medical luminaries as William Osler, John Rose Bradford, Archibald Garrod, Jonathan Hutchinson, William Hale-White, and Humphrey Rolleston too, might well have been described (as Powell was) in *Munk's Roll* as an ‘accomplished general physician’ if with specialist interests as well.^39^ Members of Neurological Society of the United Kingdom, like many of the specialist societies in London that dissolved with the advent of the Royal Society of Medicine, involved themselves from the beginning in the creation of that organisation. Hutchinson, Hale-White and Humphrey Rolleston were members of that society at the time of its dissolution. Indeed fifty-four members of the former Neurological Society joined the Association of Physicians in its inaugural year and were active participants in its proceedings until the 1920s.^40^ In short, it seems clear that many active members of the Association of Physicians and the Royal Society of Medicine would have readily agreed in 1907 with Osler's 1897 injunction: ‘that the student of internal medicine cannot be a specialist’.^41^

## Neurology in the Hospital, 1907–1946

Specialist hospitals for nervous diseases had begun appearing in Britain from the late-1850s.^42^ Many of these institutions treated conditions that had been differentiated from cases found in asylum medicine.^43^ All of these hospitals for nervous diseases and epilepsy were small institutions, and for the majority of patients with nervous conditions (often located at a great distance from these special hospitals) the likelihood of receiving in-patient or out-patient care from a specialist was slight. Almost all patients, moreover, preferred to be cared for at home in the years before the First World War.^44^ The general hospitals (old poor law and voluntary alike) and clinics were therefore the patient's most likely destination. Teaching, voluntary and old poor-law hospitals alike confronted numerous challenges with nerve patients. Primarily there was the issue of determining which cases should be brought to the attention of the specialist in nervous diseases, a rare figure.^45^ Many patients presented with conditions inclusive of nervous system involvement. Any physician capable of determining which patients should see specialists, and which should not, might well therefore have regarded him- or herself as skilled (or adept) enough to bypass the necessity of a referral to a neurologist. Furthermore, centralisation of nervous patients meant also recognising that ‘more than one-half of the persons who seek relief at the neurological out-patient department of a general hospital are suffering from functional as opposed to organic disease’. There was an obvious solution in 1922: ‘the appointment at each of the large hospitals of a resident medical officer for the department of nervous diseases.’^46^ The solution, if sensible, was by no means simple to implement. There would be very few departments of nervous diseases before 1945, and it was unlikely that most general hospitals could afford to hire a resident medical officer to differentiate cases for neurology referral only. Whoever was hired for such a job would necessarily have to be a good, young general physician—they could develop specialist interests on the job.

The organisation of the hospitals did increasingly reflect specialisation around therapeutics, laboratory diagnostics, and surgery by the close of the nineteenth century, a fact most clearly reflected in the voluntary hospitals. An Electricity Department founded at St Mary's Hospital in 1881 converted into a neurological department under Wilfred Harris in 1903. Harris thereby transformed a formerly rehabilitative service for a general medical department into a diagnostic department.^47^ His was the first department of neurology founded in Britain, and one of very few formed in the pre-First World War years. The 1912 appointment of Herbert Campbell Thomson to a special department for ‘Diseases of the Nervous System’ at the Middlesex Hospital offers another example. As Thomson explained in his history of the hospital, his appointment as a physician and lecturer in nervous diseases occurred because of undergraduates' demand for neurological teaching. Although the re-categorisation of Thomson's clients as ‘nerve patients’ represented an administrative achievement of a kind for neurologists, his primary appointment as Physician-to-Outpatients to the Hospital had not effectively ended. By not creating an in-patient service for nerve patients, the hospital administrators had only recognised officially as a specialist service a general out-patient clinic that he was providing already. Thus, aside from new teaching obligations, the position provided him with little save the ability to justify a further focus on nerve patients.^48^

Positions like those held by Harris and Thompson would typically have encompassed mental diseases as well. Thus even within worlds of emerging specialism there was an *esprit de généralité*. William Johnson's experiences in Arthur Hurst's out-patient neurological clinic at Guy's Hospital, again another example of a voluntary hospital, captures well the sensibility. Guy's Hospital had recruited Johnson in 1917. Hurst, very much cut from generalist cloth himself, required a Senior Registrar with a talent for treating neurotic patients. Johnson's practical knowledge of the psychoneurosis, gained on the Western Front, meant that he had worked with both cases of mental and nervous diseases and no doubt had seen his fair share of a wide variety of acute injuries as well. Johnson's obituarist later recalled that he had never been ‘an exclusive specialist’ and that he ‘remained one of the lessening band of general physicians, at home in all aspects of medicine and with wide practical interests’.^49^ This point was one worthy of celebration, even in 1949.

By the 1930s, many county and municipal hospitals had responded to the emergence of specialisation and begun changing their administrative arrangements as well—although this was haphazard.^50^ In 1933, Frederick Menzies, then Medical Officer of Health for the London County Council, drafted a report focusing on the appointment of specialists in London County Council hospitals. He claimed that each hospital should hire a ‘gynaecologist; ophthalmic surgeon; ear, nose and throat surgeon; orthopaedic surgeon; dermatologist; pediatrist; urologist; radiologist; obstetrician; tuberculosis officer’. Menzies demurred, however, that while neurologists would be useful: ‘… the amount of time for which he would be required is difficult to estimate, I suggest a panel of neurologists should be formed and their services should be utilised as required, and that they should be paid a fee of £2.12s.6d. per session.’^51^ Menzies' report, on one hand a bold estimation of the needs for specialist services in London, serves on the other hand as a reminder of how slow were the general hospitals adapted to specialisation. Indeed it is worth nothing that where specialist neurology departments formed they usually did so in those environments where medical teaching took place, and individuals trained in neurology otherwise held visiting appointments to county and municipal institutions.^52^

Although neurologists may have been unusually disadvantaged in these patterns, it is clear that the tendency towards general appointments remained firmly ensconced and was only incrementally reforming in London. A 1932 letter from Francis Fraser (Director of the Medical Unit at St Bartholomew's Hospital, the oldest royal charter hospital) to Alan Gregg at the Rockefeller Foundation captures the difficulties. Noting that Cecil Hinds Howell had begun a consultative neurologic clinic for out-patients at the hospital, Fraser observed that there was an otherwise hopeless situation for neurology there: ‘The plans for a neurological department failed to mature last year and will fail again this year … the formation of new special departments does not meet with approval. It is feared that such special departments must in the end mean more specialised instruction for undergraduates with further cramping of the curriculum.’^53^

These were the circumstances in all major urban environments throughout England, Scotland and Wales, and stories of the struggle to found departments of neurology or nervous diseases in hospitals abound (as they likely do for all specialist departments). In 1927, the Royal Victoria Infirmary in Newcastle appointed George Hall in neurology.^54^ In 1934, the General Infirmary at Leeds established a number of specialist departments, and in 1937 the neurologist Hugh Garland became an Assistant Physician there with an interest in Nervous Diseases, although his hospital established a neurological department only in 1947.^55^ Hospitals in Scotland and Wales were little different from those in England. The Victorian Infirmary in Glasgow had appointed a physician in nervous diseases in 1914.^56^ The Western Infirmary in Glasgow appointed its first neurologist in 1941, and Aberdeen did not have a neurological department until after the Second World War.^57^ The Edinburgh Royal Infirmary was a similarly conservative institution.^58^ In Wales a 1948 government study of specialist services observed scant neurological services.^59^ Such facts reflected the substantial role general physicians and general practitioners continued to play in treating patients with nervous diseases, both in Wales and across Britain. Indeed, even in 1960, it was an inevitable conclusion that general physicians and general practitioners almost exclusively saw patients suffering from neurological illnesses.^60^

Throughout the period prior to the formation of the NHS, the academic and clinical institutional relationships among hospitals, medical schools, universities and external examining bodies were highly contingent upon local circumstances and the training of undergraduates in bedside neurology reflected this reality.^61^ In Britain universities and hospitals faced highly differentiated circumstances, but hospital managers in particular, were suspicious, as historian Christopher Lawrence noted, of ‘academic medicine’ as ‘part of a real and realizable social order’ that was ‘professional and technocratic’ and had its ‘implicit and explicit accounts of a healthy nation’.^62^ Until the First World War, faculty in medical schools in London, throughout England, and in Scotland and Wales, thus appear to have fought the incursion of the State into their educational and clinical work. They resisted the allocation of State salaries, block grants and even the recommendations of government bodies to modernise medical training, degrees or scientific research facilities, not least because they claimed to fear the lack of autonomy such financial arrangements might bring. The formation of academic departments and specialist clinical departments run by academic staff, which became more commonplace in the interwar period, thus resulted from many different pressures, including: competition between educational bodies, falling undergraduate and graduate enrolments, recommendations by recognised governing authorities and philanthropic patrons, and even an increased valuation of specialisation.^63^

In practical terms, although not in an exclusively academic sense, specialist training, when it happened, occurred in postgraduate educational settings.^64^ By the interwar period this increasingly meant specialist training in nervous diseases happened at either the National or Maida Vale Hospitals for Epilepsy and Nervous Diseases in London. In a report drafted in 1966, a history of those hospitals explained that they finally began developing professorial units linked to the University of London in 1950:

With the advent of the National Health Service, the National Hospital was linked to the Hospital for Nervous Diseases, Maida Vale, and they are now one hospital. In 1950 the Medical School was incorporated in the British Postgraduate Medical Federation of London University as the Institute of Neurology. We were slow to alter the original internal structure of our old established Postgraduate Medical School to that of an Institute, with the establishment and growth of professorial units.^65^

Of those physicians often remembered as pioneers of neurology, many had held positions as clinical professors of medicine. Edwin Bramwell, very much in the generalist tradition of his pioneer-neurologist father Byrom Bramwell, had been appointed Moncrieff-Arnott Professor of Medicine at Edinburgh University in 1921.^66^ His retirement, however, was recognised there with the creation of a permanent Lectureship in Neurology, to which Bramwell's former Registrar, William Ritchie Russell, was appointed in 1938. Oxford University, later hired Ritchie Russell as a Lecturer in Neurology in 1949. He would eventually hold the first Chair of Clinical Neurology at the University, which was established in 1966.^67^ More broadly a collective biography of members of the Association of British Neurologists, a body formed in 1933 (and the members of whom were invited by invitation only), identifies 23 professorships held by members across their careers. Of these only five were Professorships of Neurology in Britain (a sixth was at Harvard University). The range of their professorial positions captures Britain's generalist context: five were Professors of Medicine; three Professors of Physiology; two Professors of Pathology; and one was a Professor of Morbid Anatomy, Experimental Neuropathology, Mental Pathology, Bacteriology, Neurophysiology, Neuropathology and The Practice of Medicine.^68^ Philip Cloake, Professor of Medicine at Birmingham and Arthur Stanley Barnes, Dean of the Faculty of Medicine there (both members of the Association of British Neurologists) typify these figures. They had struggled throughout the interwar period to establish a department for neurology at Birmingham. Cloake sought a tripartite division for the medical school encompassing neurology, neurosurgery, and neuropsychiatry but ultimately failed, despite being made honorary Professor of Neurology when he retired there in 1946.^69^

## The Aftermath of Generalism: Specialism in the NHS Periphery, 1946–1965

The National Health Service Act, passed in 1946 and implemented in 1948, transformed patient access to health care.^70^ Neurology was but one specialty among the 22 recognised by the Ministry of Health, and it seems probable that many specialists had experiences matching those of neurologists in the 1950s and 1960s. From the time of the creation of the health service, Ministry officials had recognised the need to increase the number of practising neurologists (as well as all specialists). In 1948, they had thus presented ‘general physicians with an interest in neurology’ as a temporary necessity—the qualification itself reveals the transformation bureaucratisation brought to the practice of medicine. Ministry officials also recommended that between 100 and 150 beds per million of population be reserved for neurological patients and declared that each regional centre should provide a neurological service.^71^ Under the new legislation, the Ministry of Health managed the health service through a hierarchical system of Regional Hospital Boards (four for the London area; 12 for other regions of the country).^72^ Ministry officials, frequently criticised in the press for over-centralisation, increasingly adopted a hands-off approach and thereby empowered members of the Regional Hospital Boards to undertake local management of health care. By 1953, according to neurologist Walter Russell Brain, then President of the Royal College of Physicians London, the Ministry had significantly diminished its advisory role, especially towards the Regional Hospital Boards. These facts meant that by the mid-1950s medical specialists, especially in non-surgical specialties, while better represented in teaching centres and major urban environments, found it difficult to find employment beyond the teaching hospitals and most metropolitan-bound general hospitals.^73^ Specialists' difficulties, according to figures at the Royal Colleges, derived from policy tendencies that had become increasingly exaggerated by the mid-1950s. General physicians commonly controlled the Regional Hospital Boards and preferred to hire physicians with some training in specialist subjects rather than exclusive specialists. Some observed also that members of Regional Hospital Boards preferred to hire specialised surgeons over specialised physicians, because, in the words of Dr George E. Godber (one of the major architects of the health service), surgical specialists could supposedly ‘kill two birds with one stone’ through diagnosis and subsequent intervention.^74^

Some clinical neurologists viewed this situation as a crisis. In a series of reports published by the Committee on Neurology of the Royal College of Physicians London, neurologists endeavoured first to understand and then secondly steer the future development of neurology within the health service. Committee members in the early 1950s, for example, were shocked to discover that the Ministry of Health recognised officially only 38 neurologists for the whole of the country.^75^ Further details supplied by the Ministry of Health, as well as hospital and patient data gathered mainly from London hospitals, added to what they deemed a bleak picture for the state of British neurology.^76^ Importantly, most physicians and medical administrators who reviewed the Committee's 1954 report, if not agreeing with the neurologists' solutions, nonetheless concluded that their report was a fair representation of the circumstances for all medical specialists beyond the teaching hospitals.^77^ It seems that hiring practices within the Regional Hospital Boards benefited general physicians with some training in a specialty rather than pure specialists.

The Committee on Neurology continued to study the organisation of neurology in Britain throughout the 1950s and 1960s and continued also to recommend that general physicians with an interest in neurology be considered an obsolete occupational category.^78^ In the early 1960s and as part of that campaign, the Committee surveyed 86 neurologists working across Britain about the circumstances of their labour (see Table 1). It is important to note that prior to sending out the survey, the committee members adopted the position that most neurologists were working without adequate beds, serving populations that were too large, had poor access to research facilities, and were largely dissatisfied with their work. In that sense, the survey, which was never tabulated, had its conclusions already baked in. Yet while the results from this survey were never used because they had proved ‘too complicated to collate’, analysis now of the questions in the survey and the neurologists' answers offers a fascinating picture of neurological practice under the NHS.^79^ For the purposes of this essay, perhaps the most interesting question to which the Committee sought answers was whether general physicians with interests in neurology were useful or could be usefully employed—examined below.^80^

**Table 1.** Physician, Region, Assessment of Research Facilities and Relationship with University

Name	Region	Research facilities available to you	Relationship with university or research centre
Henson, R. A.	NEM		
Ashby, M. G. C.	NWM	Excellent	Good
Bates, J. A. V.	NWM	Good	mrc staff- inside
Brinton, D. H.	NWM	National Hospital	Inside
Carter, A. B.	NWM	Poor	Excellent
Dimsdale, Mrs. H.	NWM	Good	Inside
Gilliat, R. W.	NWM		Inside
Gooddy, W. W.	NWM	Good	Good
Hulbert, N. G.	NWM		
Jewesbury, E. C. O.	NWM	Good	
Marshall, J.	NWM		Inside
Milnes, J. N.	NWM	Use limited by time	Poor
Nathan, P. W.	NWM	Excellent	Inside
Parsons-Smith, B.	NWM	Good	Inside
Porter, R. J.	NWM		Excellent
Sandifer, P. H.	NWM	Good	
Thomas, P. K.	NWM		Inside
Williams, D. J.	NWM	Excellent	Excellent
Hierons, R.	SEM	Poor	Poor
Foley, J.	SWM	None	None
Kendall, D.	SWM	Poor	Not close with university, fairly close with A. Morley- none
Rose, F. C.	SWM	Poor	Research dept. at RCS- inside
Small, J. M.	SWM	None	None
Foster, J. B.	1	Excellent	RNC- inside
Miller, H. G.	1	Good	Inside
Walton, J. N.	1	Excellent	Excellent
Astley, C. E.	2	None	Excellent
Cook, J. B.	2	Poor	Inside
Espir, M. L. E.	3	None	None
Matthews, W. B.	3	Poor	None
Guttmann, L.	9	Good	Inside
Spalding, J. M. K.	9	Good	Excellent
Whitty, C. W. M.	9	Poor	Excellent
Alcock, N. S.	SW	None	None
Russell, W. R.	9	Good	Inside
Campbell, A. M. G.	SW	Good	Poor
Wilson, T. G.	SW	None	None
Bickerstaff, E. R.	12	Good	Good
Guest, I. A.	12		
Holmes, J. M.	12	Good	
Jefferson, J. M.	12	None	Excellent
Gordon, N. S.	13	Good	Inside
Slatter, K. H.	14	Excellent	None
Graveson, G. S.	15	Excellent	None
Lloyd, G. H. T.	11	Good	Poor
Rees, W. E.	11	Poor	Poor
Spillane, J. D.	11	Poor	
Wells, C. E. C.	11	Poor	Excellent
Simpson, J. A.	4	Poor	Good
Stanton, J. B.	4	Poor	Excellent
Hall, G. S.	12	Queen Elizabeth Hospital	
Croft, B. P.	NEM	Good	Inside
Hughes, R. R.	14	Good	Poor
Stewart-Wallace, A. M.	SEM	Good	None
Kelly, R. E.	NWM	Poor	Good
Heathfield, K. W. G.	NWM	None	Good
Ironside, R. N.	NEM	Good	

Among the many surprising details revealed by their survey was the population size each neurologist served. Some neurologists reported extreme windows. Helen Dimsdale, for instance, noted that there were 4 million people in her region, North Western Metropolitan. That region, the survey reveals, was served by 16 neurologists. By contrast, J. B. Cook and C. E. Astley, working in Region 2, alone provided a neurological service to 3.5 million. Another way of measuring the impact serving such a large population had on these neurologists was the numbers of miles they reported driving. Eight reported driving over 800 miles per month. In the extreme, G. H. T. Lloyd, in Region 11 claimed to average 2,300 miles per month. Most neurologists reported having beds for their patients, but most also claimed that the supply of available beds was inadequate for demand. Many neurologists reported no difficulty finding replacements for sickness or holidays. A few, however, reported ‘no covers’ and ‘inadequate’ assistance.

**Figure 1.**
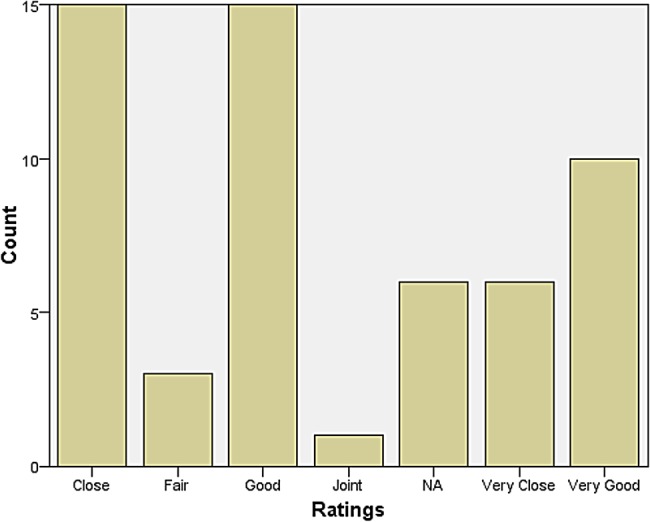
Relationship with Neurosurgery.

**Figure 2.**
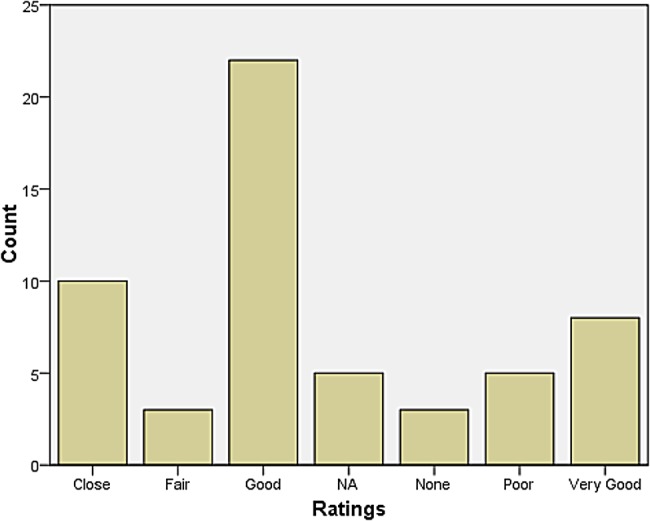
Relationship with Psychiatry.

**Figure 3.**
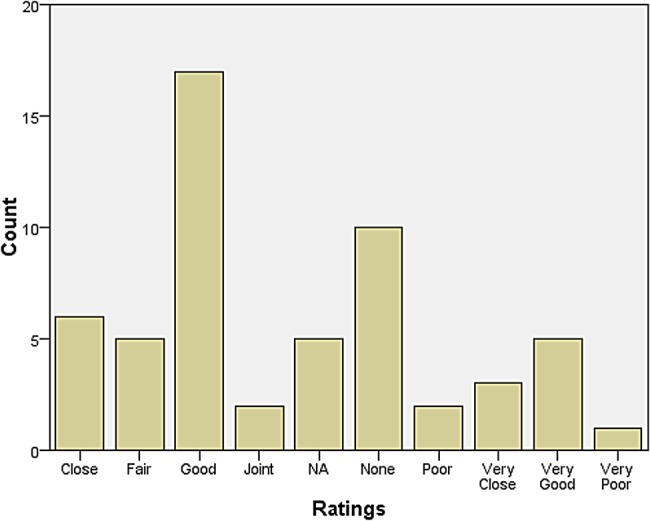
Relationship with Neuropathology.

**Figure 4.**
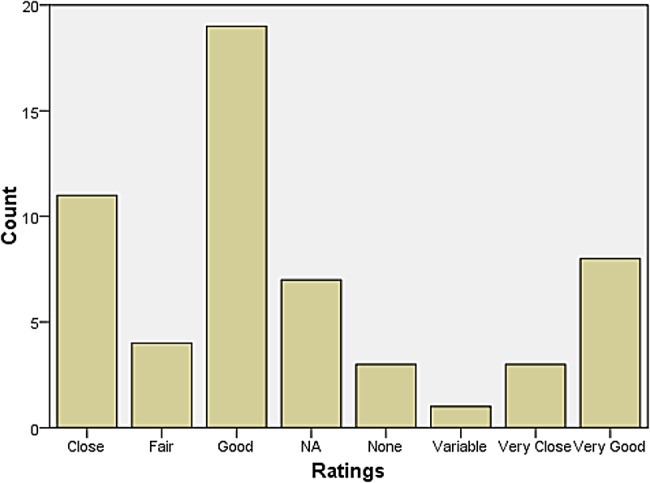
Relationship with Gen Med and Surgery.

**Figure 5.**
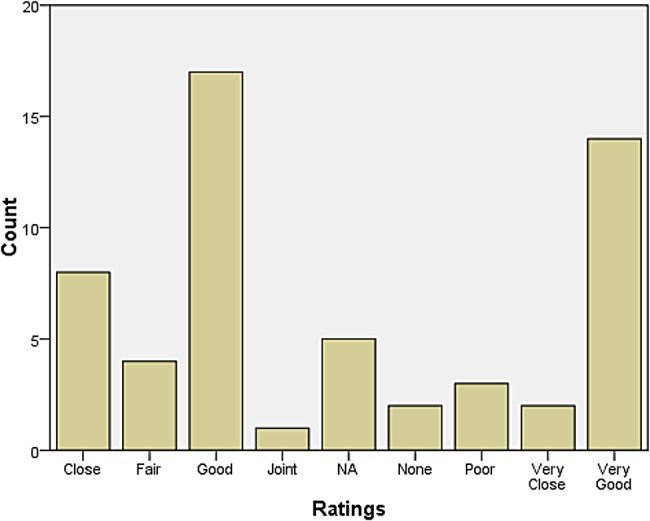
Relationship with Neuroradiology.

**Figure 6.**
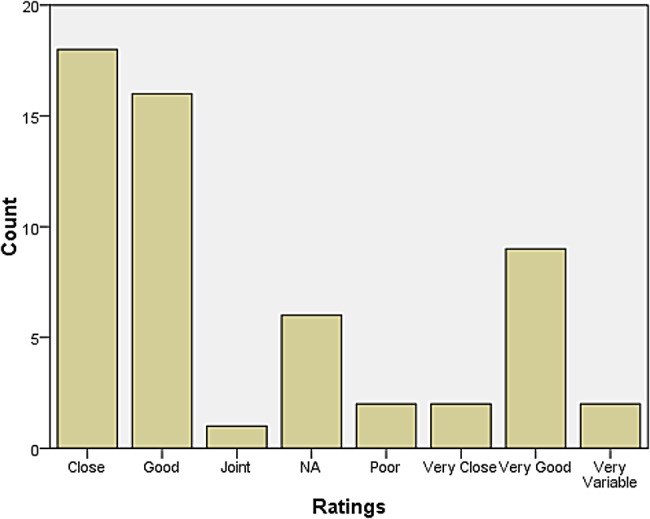
Relationship with E.E.G.

A rather larger group declared themselves to be at some distance or even remote from other neurologists. Those who reported feeling isolated also tended to report inadequate research facilities or connection to universities. When asked how they evaluated their relationship with colleagues in neurosurgery, most responded that relations were ‘very good’, ‘very close’, ‘good’, and ‘close’. The same was largely true for psychiatry, neuropathology, and general medicine and surgery (see Figures 1–4). Relations with neuroradiological and EEG departments were somewhat more strained, but this fact was likely due to the limited presence of those autonomous departments across the country (see Figures 5–6).

The one significant inferential statistical finding that can *now* be derived from the survey data came in the answers to the question about whether or not general physicians with interests in neurology were available and helpful. When respondents did indicate the presence of one or more general physicians with interests in neurology then 70 per cent of them indicated that they found that relationship advantageous. Fully 90 per cent of those without such a relationship, in sharp contrast, imagined it would be of limited advantage (Table 2). Such a striking disparity in response probably reflected several factors: the size of the population served, local resources, travel, access to other neurologists, professional relations with local psychiatrists and proximity to London.

**Table 2.** General Practitioner is Useful X General Practitioner is Present Crosstabulations

			GP Present		
			No	Yes	Total
GP useful	No	Count	19	8	27
		% within GP Pres	90.5%	29.6%	56.3%
	Yes	Count	2	19	21
		% within GP Pres	9.5%	70.4%	43.8%
Total		Count	21	27	48
		% within GP Pres	100.0%	100.0%	100.0%

* In addition, we calculated Pearson Chi-square statistics which indicated the crosstabulation results were spastically significant (P < 0.000; result not presented in Table).

## The aftermath of specialisation. Comparative thoughts for a post-disciplinary age

At a 1952 general meeting of the Association of Physicians the President, seizing upon the mission of the Association to promote ‘internal medicine’, concluded his Presidential Address:

It seems to me therefore that the Association has great opportunities ahead and an important work to do. I suggest that the interpretation of the duty of promoting Internal Medicine should be to evaluate all the new material after it has been perfected in the laboratory and tested by the specialist to whom it more particularly applies and assess its place in General Clinical Medicine.^81^

On one hand, we might interpret such a statement now as mere posturing, the rhetorical grandstanding of a stakeholder group of specialists endeavouring to claim a particular jurisdiction. On the other hand, we might see such a statement as conjuring a generalist attitude that had remained very much an omnipresent reality of British medicine from the Victorian period through to the Cold War Era. Of course, as the President's Address makes clear, no one was really questioning the validity of the specialisation process *per se*. Nonetheless, the oft-alleged inevitability of specialisation was not accepted by everyone, and some regarded the utility of specialisation in terms of the higher ideal of general integration. The case of neurology makes this unusually explicit—just in the way that the case of ophthalmology, by corollary, makes the advance of specialism equally obvious.^82^ These tensions in neurology continued well into the twentieth century. As late as 1984, one professor of medicine opined in the *British Medical Journal* that neurologists failed when they made their work an exclusionary activity. That professor's highly controversial remarks, however, were aimed more widely than neurology alone. Wanted, he argued, were physicians with rich and wide backgrounds for a variety of specialist purposes. A general training, he proffered, was the one more suited for the realities of medicine.^83^

The curious feature of much historical scholarship on medical specialisation is the way that writers assume both inevitability and ubiquity.^84^ At the time when this essay was written, when people talked about medical specialisation, they attached a sense of finality to the process that suggested it was an end to itself. Such views do raise an interesting dilemma about the progress of medical knowledge, for in periods different from our own, the idea appears to have been that expert discoveries, whether in medicine or surgery, would inform, improve and shape a general knowledge. This was an epistemological position. Perhaps equally curious, then, is where this ubiquity and inevitability *was not*. At the close of the twentieth century, there was a growing movement in the modern university that reflected on the traditional disciplines through multi-disciplinary, trans-disciplinary and even anti-disciplinary lenses. While discipline formation and specialisation need not be construed as precisely equivalent sociological processes, the fact that we can ‘un-think’ discipline formation but not specialisation underscores a poignant reality about the function of social categories in the social history of medicine. In other words, it had become strangely easier to imagine a return to general education in the modern university but practically impossible to imagine a reinvigorated ‘generalism’ once again in medicine.^85^ Whether such a disconnection is alienating falls beyond the scope of this study, but it does suggest that there are literatures from the 1970s about labour and medicine worthy of revisiting and perhaps with a somewhat more sympathetic eye to the value that studies of medical labour might bring to the historiography of medicine.^86^

But perhaps, too, there are signs pointing in a different direction—here circumstances in neurology in the contemporary United States may well suggest a prospective future. Representatives for the American Academy of Neurology in the comparatively recent past had begun lobbying to have neurology recognised by the US government for primary care incentives under Medicare (which American neurologists can only do if they are accorded primary care status).^87^ The neurologists' argument, which on the surface appeared mainly about remuneration, was actually about the role of cognitive evaluation in primary care. Figures from the American Academy of Neurology claim that because one in six patients in primary care settings require cognitive evaluation (often stroke patients), they, the neurologists, were therefore the right group to do those procedures and that under the present arrangement the federal government of the United States was being unfairly stingy and anti-progressive. It is interesting to speculate about the resulting consequences should either the US government or primary care providers adopt an approach similar to that found in the UK in the past. Advocates for primary care practitioners in the USA could certainly argue that if they received more training in basic neurology, then they could thereby supplant more expensive neurologists in primary care settings. The American Academy of Neurology has also recently noted a decline in the number of neurologists per capita in the USA. As this study has demonstrated in the case of Britain, it was a continual shortage of specialist labour that ultimately justified training and hiring general physicians with an interest in neurology. That shortage of specialist labour derived primarily from a potent faith, one grounded in an epistemological perspective, in the value of enduring generalism for medicine.
